# Description of a personality syndrome in a common and invasive ground beetle (Coleoptera: Carabidae)

**DOI:** 10.1038/s41598-018-35569-z

**Published:** 2018-11-30

**Authors:** Sophie Labaude, Niamh O’Donnell, Christine T. Griffin

**Affiliations:** Department of Biology, Maynooth University, Maynooth, Ireland

## Abstract

Animal personality, defined as consistent differences among individuals in their behaviour, is being increasingly studied as it might lead to a new understanding of the evolution of behaviours. Despite a clear interest in studying personality in a wide range of taxa for comparative analyses, studies on invertebrates are still scarce. Here, we investigated the personality of a ground beetle, *Nebria brevicollis*, which is widespread in Europe and invasive in North America. We measured seven behavioural traits from an array of three different tests: (i) activity and exploration related traits; (ii) reaction to a threat, and (iii) phototaxis. The repeatability was tested by measuring all behaviours twice, on different days. All behavioural traits were consistent through time, highlighting the presence of personality in the beetle. In addition, we analysed the relationship between the different traits and highlighted two clusters of behaviours (behavioural syndrome), one grouping activity, exploration and boldness traits, and a second one consisting of responses to a threat. This study is the first to our knowledge to provide evidence for personality dimensions within the vast group of the Carabidae. It also constitutes a preliminary step in the experimental investigation of the importance of animal personality in invasive species.

## Introduction

It is now well established that the understanding of the evolution and maintenance of animal behaviours requires, in addition to their average responses as individuals or groups, to also take into consideration inter-individual variations in their behaviour. In this respect, a strong interest has been demonstrated over the last two decades regarding the study of animal personality. Animal personality can be defined as consistent differences in the behaviour of individuals across time and situations^1^. Clusters of behaviours that correlate in a given context or between multiple contexts define behavioural syndromes^1,2^.

Despite invertebrate species representing over 95% of all animal species^3,4^, personality studies have traditionally focused on vertebrate species^5^. Indeed, because of their higher cognitive abilities, vertebrates were considered as more likely to exhibit complex behaviours while invertebrates were thought to behave stereotypically, thus showing few or no individual differences in their behaviour. Lately, substantial efforts were made to include invertebrates in personality research (see Kralj-Fišer & Schuett^6^; Mather & Logue^7^ for reviews). These studies were mainly focused on a few taxa in molluscs and arthropods. In particular, cephalopods which are considered as advanced invertebrates in terms of cognitive functions have been more thoroughly studied in this respect than most groups of invertebrates^8–10^. However, it is being increasingly recognised that the study of personality in invertebrates is of particular interest, as compared to that of vertebrates, given their ecology and biology. In addition to being easy to rear and to maintain in laboratory conditions, their often short life cycles make them a good study model. This is especially true in the case of genetic studies, where invertebrates can be used for the observation of the transmission of personality traits through several generations. Their simpler nervous systems, compared to vertebrates, are also of great value in the understanding of the neurobiology and physiology underlying variations in personality. Finally, unique life-history features also make invertebrates good models to study personality and its evolution (both phylogenetic and ontogenetic), such as asexual reproduction or metamorphosis^6,11^. Despite their interest, there is still a lack of representation of most invertebrate taxa in personality studies. As pointed out by Kralj-Fišer & Schuett^6^, this makes comparative analyses impossible to conduct, although such analyses taking into account numerous and diverse vertebrate and invertebrate taxa would shed a new light on the evolution of personality. In addition, the study of personality in invertebrate could provide a better understanding in other fields, such as population and community ecology^11,12^. Recent studies also suggest that personality could affect species distributions, species invasions and response to environmental changes^1,13^.

Among insects, some species have received substantial attention such as crickets (e.g. Kortet and Hedrick^14^; Wilson *et al*.^2^; Niemelä *et al*.^15,16^) or social insects (see Jandt *et al*.^17^ for a review). On the other hand, compared to their relative importance, only a few studies exist so far about behavioural repeatability in Coleoptera, the largest order of insects representing close to 40% of insect species and a fourth of all animal species^4^. These studies often focus on death-feigning behaviour^18,19^ or activity levels^20,21^. For instance, seed beetles *Callosobruchus maculatus*^21^ and red flour beetles *Tribolium castaneum*^20^ showed behavioural repeatability over time in several activity-related traits. Because such traits could be related to specific selection pressures linked to stage-dependant ecological needs, such as dispersing or mate-searching in adults, Wexler *et al*.^20^ suggested that more diverse traits, such as phototaxis or threat-linked behaviours, should also be examined. Indeed, behaviours might not evolve independently but rather as packages of several traits, leading to correlations between different behaviours defining behavioural syndromes^1,2^. Investigating such behavioural syndromes among many taxa is thus particularly relevant to better understand the selective forces shaping animal personality. In addition, the ecological and evolutionary importance of this concept has been highlighted in several recent papers^1^. For instance, the selection of two opposite behavioural syndromes over generations was found to depend on the intensity of competition in birds^22,23^. However, few empirical studies investigated multiple behavioural dimensions in insects so far (e.g. Wilson *et al*.^2^; Gyuris *et al*.^24^). Such studies remain even more scarce in Coleoptera. For instance, Tremmel and Müller^25^ performed a battery of five different behavioural tests on mustard leaf beetles (*Phaedon cochleariae*), leading to the description of three personality dimensions: boldness, activity, and nontargeted explorativeness. They also showed that the personality of individuals was partly dependant on environmental factors: beetles fed with a low-quality diet proved to be bolder but less active than individuals fed with high-quality food, while this parameter did not influence their explorativeness^25^. Monceau *et al*.^26^ tested four behavioural traits in mealworm beetles (*Tenebrio molitor*) and also found most of them to present repeatability within the same developmental stage, with certain traits being also correlated to each other (such as activity and exploration or food neophobia and exploration), thus revealing a behavioural syndrome. In addition, they highlighted differences in behaviours and in their repeatability between larvae and adults^26^ (see also Løvlie *et al*.^21^).

In this study, we investigated the personality of *Nebria brevicollis*. This carabid beetle is one of the most common and widespread ground beetles in Europe^27,28^. It has recently been detected in several places in North America, where it has been identified as an invasive species^29^. Given its ecological importance, understanding its behaviour might shed a new light on the implication of personality in problematics linked to the community ecology of widespread species, and might also be useful in the study of traits linked to invasion success^11,30,31^. Moreover, although carabids represent a large and widespread group, accounting for more than 40,000 species worldwide^32^, no research has been conducted on their personality to our knowledge. We performed three different behavioural tests, from which seven behavioural traits were measured. In addition to traits related to activity and exploration, two widely studied parameters in animal personality^33^, we also included phototaxis and reaction to a threat, following the suggestion made by Wexler *et al*.^20^. All behavioural traits were measured twice, with 24 h to several days between trials, in order to investigate the repeatability of each behaviour through time. The associations between the different behavioural traits were also studied to identify possible personality dimensions.

## Results

**Figure 1:**
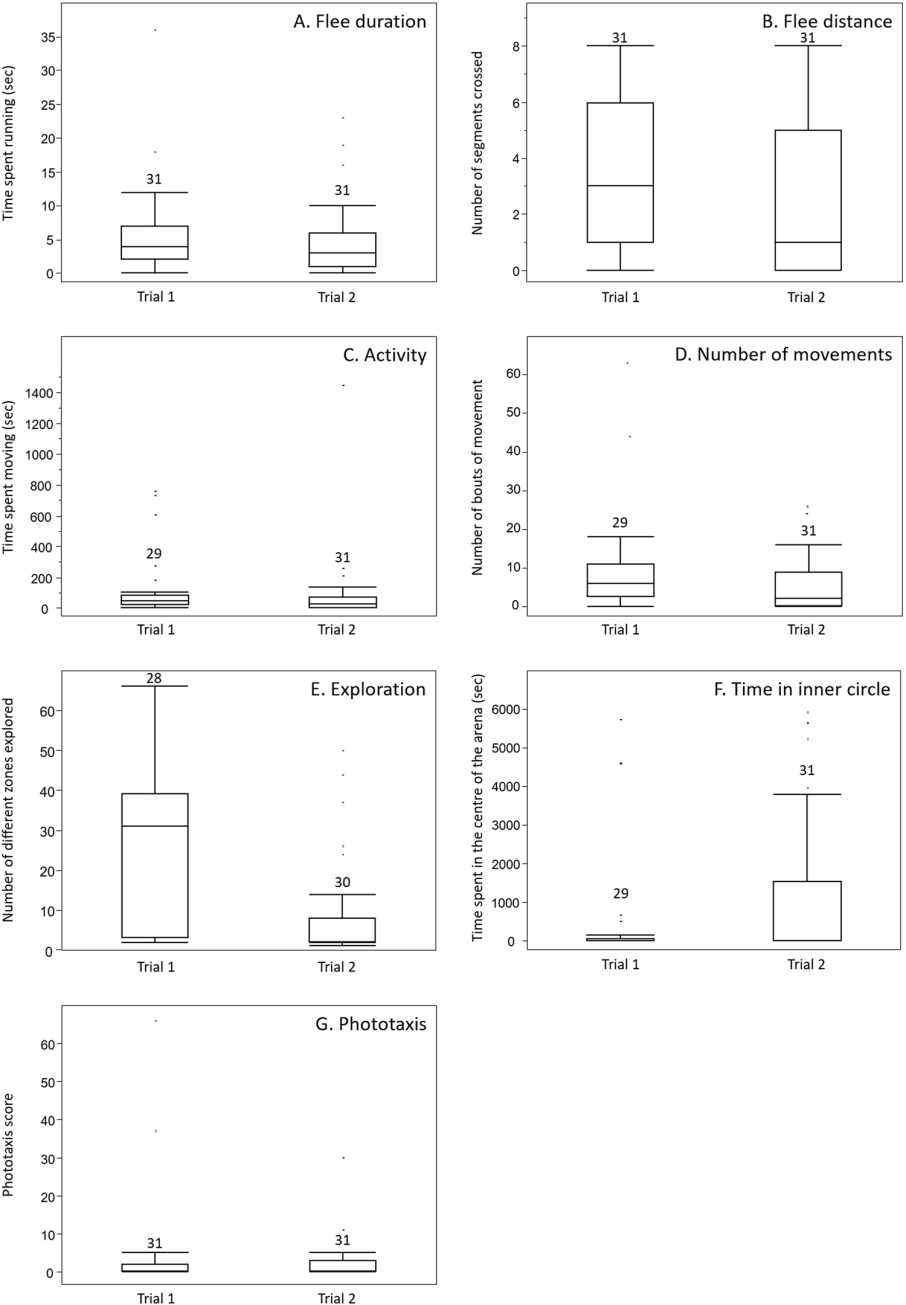
Boxplots of the different behavioural traits measured in *N. brevicollis* beetles. Each test was done twice (trials 1 and 2). The first test investigated the reaction to a threat as the time (**A**) and distance (**B**) spent running after a mechanical disturbance. The second test investigated activity and exploration, with the time (**C**) and the number of bouts of time (**D**) spent moving over 90 minutes, the number of different zones crossed in a total of 69 zones (**E**) and the time spent not touching the arena walls (**F**). In the last test, the phototaxis score, ranging from 0 to a maximum score of 120, was measured (**G**). Boxplots show the median and quartiles, and dots above each boxplot indicate outliers.

**Figure 2:**
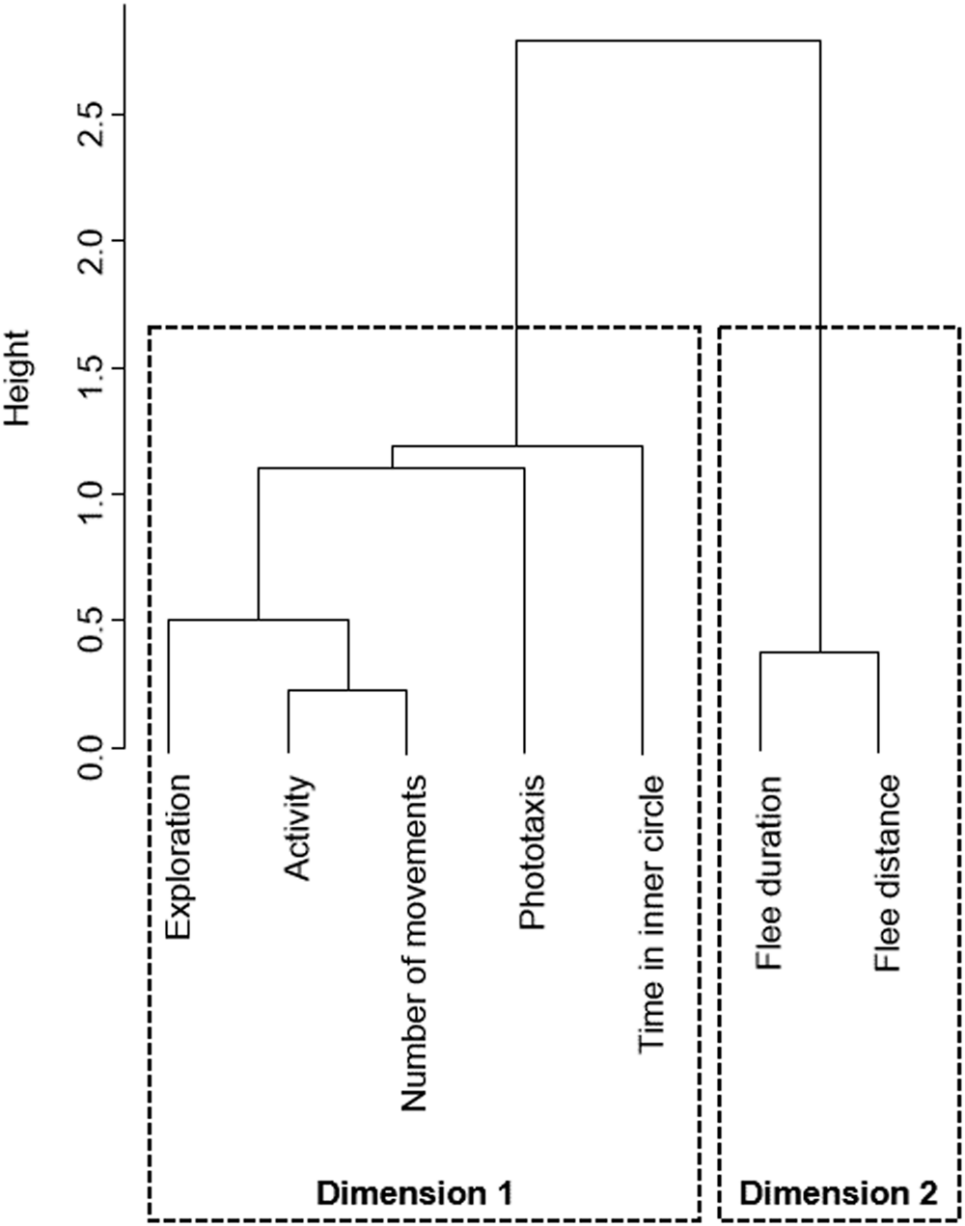
Dendrogram showing the relationship between seven behavioural traits investigated in the ground beetle *Nebria brevicollis*. Two personality dimensions were identified using an agglomerative cluster analysis. The height indicates similarity based on the absolute values of the Spearman correlations among the behavioural traits: the shorter the distance, the more similar the variables are to each other.

Overall, beetles showed a low activity and exploration, as well as a high photophobic behaviour (Fig. 1). However, inter-individual variation was found in all the traits (Fig. 1). Although the results varied between trials (Fig. 1), the ranking of the insects remained similar. Indeed, all the seven behavioural parameters tested (flee duration, flee distance, activity, exploration, number of movements, time in inner circle and phototaxis) showed a significant repeatability between the two successive tests (Table 1), highlighting their consistency over time. An agglomerative cluster analysis revealed two groups of behavioural traits (Fig. 2). The first personality dimension comprised five traits: exploration, activity, number of movements, phototaxis and time in inner circle. The second personality dimension comprised the two behaviours linked to threat reaction: flee duration and flee distance. Within each dimension, all behavioural traits were significantly correlated between each other, except for the time in inner circle for which the correlations with phototaxis and exploration were not significant (Table 2). No significant correlations were found between the behaviours belonging to the two different dimensions.

**Table 1:** Spearman correlations between the two tests of each behavioural trait measured in the ground beetle *Nebria brevicollis*.

Test	Behavioural trait	Spearman r	p-value
Reaction to a threat	Flee duration	0.60	0.0004
	Flee distance	0.59	0.0005
Activity and exploration	Activity	0.56	0.0017
	Number of movements	0.47	0.0099
	Exploration	0.54	0.0034
	Time in inner circle	0.69	<0.0001
Phototaxis	Phototaxis	0.64	0.0001

**Table 2:** Spearman correlations between the different behavioural traits (average of the two trials for each trait) within each personality dimension found in the ground beetle *Nebria brevicollis*.

Trait 1	Trait 2	Spearman r	p-value
**Dimension 1**			
Exploration	Activity	0.74	<0.0001
Exploration	Number of movements	0.81	<0.0001
Exploration	Phototaxis	0.51	0.0035
Exploration	Time in inner circle	0.30	0.099
Activity	Number of movements	0.95	<0.0001
Activity	Phototaxis	0.49	0.0054
Activity	Time in inner circle	0.52	0.0026
Number of movements	Phototaxis	0.48	0.0066
Number of movements	Time in inner circle	0.52	0.0025
Phototaxis	Time in inner circle	0.25	0.17
**Dimension 2**			
Flee duration	Flee distance	0.80	<0.0001

## Discussion

Our study investigated seven behavioural traits of the common and invasive ground beetle *Nebria brevicollis*, measured in three different tests relative to exploration, activity, phototaxis and reaction to a threat. All traits measured in our study showed a significant repeatability through time. We thus provided evidence for the presence of personality in this species according to the definition by Sih *et al*.^1^. In addition, we found the presence of a personality syndrome, with the description of two personality dimensions. The first dimension comprised behaviours related to activity, exploration and phototaxis, with five traits measured in two different contexts. The second dimension concerned responses to a threat, two traits measured within the same test setup.

General activity, both in terms of time spent moving and number of movements, is an important trait in the field of personality that, along with exploration, often presents positive correlations with traits related to boldness (e.g. Moretz *et al*.^34^; Stanley *et al*.^35^). Although less often investigated in personality studies, phototaxis can be associated with both exploration and boldness personality axes. Indeed, individuals leaving dark places might be more exposed to potential threats, such as predators. Similar to our results, Pamminger *et al*.^36^ also found that the most photophilic ants were the most active individuals^36^. Such correlations between phototaxis and activity were also observed in amphipods^37^. The time spent in the inner circle of the test arena is representative of the edge preference (or centrophobism), a behaviour that is also commonly associated with exploration and boldness^38,39^. Indeed, similar to phototaxis, individuals leaving the security of the edges to explore the centre of the arena might be more exposed to threats. The positive correlation found between activity/exploration and boldness could be simply explained by the fact that individuals might prefer safe places when they are not moving. The most explorative/active individuals are thus the most likely to end up in a wide range of places, including those exposed to potential threats. Although such positive correlations are not systematic (for instance, Tremmel and Müller^25^, found the opposite in mustard leaf beetles *Phaedon cochleariae*), boldness, activity and exploration are commonly correlated together in personality syndromes, in both invertebrates and vertebrates (e.g. Cote *et al*. 2010^30^).

The description of the boldness/activity/exploration dimension in *N. brevicollis* might be helpful to understand its dispersion and potential invasion in North America^29^. Our study is one of the few that examined a behavioural syndrome in an invasive species^40–42^. However, several studies highlighted that the dispersal propensity of individuals can depend on personality traits such as boldness, exploration and sociability^24,30,40,43^. Because different traits can be necessary for a species to successfully go through the different stages of the invasion process, Fogarty *et al*.^31^ hypothesized that a population with a variety of life-history and personality traits would be more likely to become invasive. Using a simulation model, they indeed found that a species was more likely to spread rapidly if it included a mix of different personality types, in particular those linked to dispersal tendencies in a density-dependent way^31^. Such personality-dependant dispersal can then affect population dynamics and invasion processes. In addition, environment can affect personality. For instance, Tremmel and Müller^25^ showed that the food quality experienced by mustard leaf beetles during their development affected both their activity and boldness. This might help to understand the dynamics of species invasion in habitats differing in some environmental parameters. In addition to comparisons of the dispersal ability between closely-related invasive and non-invasive species^44,45^, investigating the variation of behavioural patterns in a given invasive species might prove particularly relevant. In this regard, comparing personality syndromes of native European and invasive North-American populations of *N. brevicollis* would shed a new light on the implication of personality in invasion processes.

The second behavioural dimension described in our study concerns the reaction to a threat. Here, contrary to most studies about the behaviour of beetles, we investigated two traits related to escape behaviour. Indeed, although the reaction of beetles to threats has been well studied, death-feigning behaviour received much more attention compared to fleeing behaviour. Although death-feigning was sometimes observed during the maintenance of our *N. brevicollis* individuals, preliminary experiments showed that beetles from this species were more likely to flee when faced with a threat, such that inducing this behaviour in a simple and repeatable way was not possible here. Death-feigning, also referred to as thanatosis, quiescence, or tonic immobility, is a common response of insects to external stimuli (e.g. Miyatake 2001^46^; Hozumi and Miyatake^47^; Honma *et al*.^48^). This behaviour has been shown to be adaptive, increasing the survival of red flour beetles (*Tribolium castaneum*) exposed to a spider predator^49^. However, fleeing also constitutes an important part of beetles’ reaction to a threat. The two strategies, fleeing or death-feigning, often negatively correlate^50^. The choice of one strategy over the other one might depend on several factors, including environmental parameters^51,52^. Interestingly, death-feigning behaviour was shown to be correlated with other personality traits. For instance, death-feigning behaviour was included in the “boldness” personality dimension in mustard leaf beetles, along with the reaction to novel objects or refuge emergence^25^. In the confused flour beetle (*Tribolium confusum*), this trait was linked to locomotor activity^18^. Although highly consistent through time, representative of a personality trait, we found that the reaction to threat in terms of fleeing behaviour did not correlate with any of the other traits that we measured. Several studies suggested that activity and death-feigning behaviour rely on similar mechanisms and genetic factors^18,19,21,53^. Here, the absence of correlation between fleeing behaviour and other traits might suggest that, contrary to death-feigning, other mechanisms are involved. Further studies are thus necessary to understand the genetic and mechanistic basis of this personality dimension. For instance, in a similar way that Nakayama *et al*. (2010) studied the genetic and mechanistic origin of death-feigning behaviour, artificial selection of individuals based on their fleeing response might be used to investigate its link with other personality traits and its genetic and mechanistic basis.

The present study demonstrates the presence of a personality syndrome in a common and invasive beetle. It is the first time to our knowledge that personality is described among carabids. Contrary to many studies working on individuals reared in the laboratory, the results obtained here concern wild individuals. Consequently, some of the variation in behaviours might be explained by traits that could not be controlled, such as inter-individual differences. In particular, the exact age and the sex of individuals could not be determined here. Although it cannot be excluded that the age of individuals could play a role, important differences in personality traits are more likely to occur between different developmental stages, as it was showed in other species^20,26^. Similarly, several studies focusing on beetles showed no differences of behaviour between sexes^18,20,25,26^. Despite variations due to uncontrollable parameters, studying wild animals leads to a better representation of the real variability in behaviours compared to laboratory-reared animals for which undesirable artificial selection can happen. In addition to the perspectives that we suggested about the integration of personality in the study of invasion, as well as the investigation of mechanistic and genetic origins of the personality dimensions, future studies might focus on other traits. For instance, sociality and aggressiveness toward conspecifics could give interesting results given that this species is often found in high densities in the wild. Considering the consistency of behaviours over longer periods of time might also be useful to better understand the ecological and evolutionary origins of personality, in particular with the investigation of the maintenance of behavioural dimensions over the life-cycle of the beetles.

## Methods

### Beetles sampling and housing

Ground beetles (Coleoptera: Carabidae) were collected during summer 2017 using pitfall traps located in a lightly wooded area in Maynooth, Ireland (Campus of Maynooth University, 53°22′54.8″N 6°36′11.9″W). Traps were checked every 2–3 day and all individuals were immediately brought back to the laboratory. Directly following each sampling, individuals from the species *Nebria brevicollis* were identified based on morphology. They were then housed in large boxes (510 × 350 × 170 mm) containing 20 mm of soil (multipurpose compost, Westland) and egg boxes as shelters. Dead *Galleria mellonella* larvae were provided *ad libitum* as food, and soaked cotton wool was given for water. A high degree of humidity was kept by regularly spraying boxes with tap water. The room was maintained at 24 °C and under a photoperiod of 14:10 light:dark. Individuals were acclimatized to laboratory conditions for several weeks before behavioural assays.

Prior to the experiments, 31 beetles were transferred into individual ventilated plastic boxes (80 mm diameter, 60 mm height) containing a thin layer of soil and a 1.5 ml Eppendorf tube filled with wet cotton. Other conditions remained the same, as explained above.

### Reaction to threat

**Figure 3:**
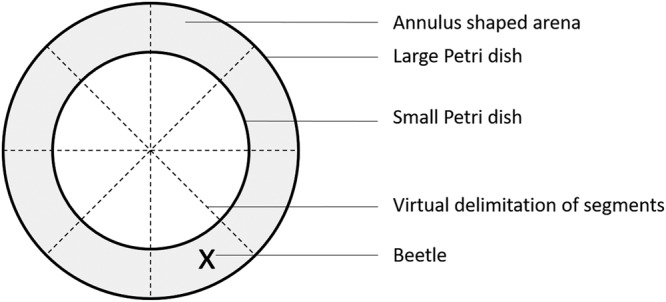
Experimental device used to assess beetles’ reaction to a threat. Single individuals were placed in the grey area. The large Petri dish was closed with a lid.

The fleeing reaction of individuals to a threat was measured using an annulus-shaped arena. The device consisted of a 90 mm diameter Petri dish with a smaller Petri dish (60 mm diameter) attached in the middle preventing individuals to walk in the centre of the arena, thus forming a 30 mm wide annulus (Fig. 3). Lines were traced under the device, intersecting in the centre, such that the arena was virtually divided into eight even segments. Individuals were kept from escaping from the device with a lid in which a 20 mm diameter hole was pierced.

Beetles were individually placed in the device and allowed to habituate for several minutes to the device until they stopped moving. The position of the lid was then adjusted so that the hole was above the individual. The fleeing behaviour was then induced by a mechanical disturbance: the beetle was gently hit on its back (end of the abdomen) through the hole using forceps. The time spent running as well as the number of segments crossed by the beetle were recorded, respectively referred as “flee duration” and “flee distance”. The trial ceased once the insect stopped moving. To assess the repeatability of the behaviours through time, the experiment was conducted twice for each individual, with 24 h between the two trials. Such time interval between the tests allows the observer to assess the repeatability of the measurements within the same developmental stage and is thus frequently used in behavioural studies^18,20,26^. In addition, testing individuals over only two trials prevents an habituation to the behavioural assay that might lead to a decrease in their responsiveness^54^.

### Activity and exploration

The activity of beetles was tested during their regular dark hours. Single individuals were placed into Petri dishes (90 mm diameter). To minimize the disturbance, the transfer was done under a red light. After five minutes of habituation, the behaviour of individuals was recorded for 90 minutes in the darkness using an infrared night vision camera (Besteker HDV-301STR). Although initially developed for vertebrates^55^, such open-field tests are now commonly used to assess activity levels and exploration in invertebrates (e.g. Kortet and Hedrick^14^; Wilson *et al*.^56^), two widely measured traits in the field of personality^33^. Resulting videos were analysed using BORIS software version v.5.1.3. (Behavioral Observation Research Interactive Software, Friard & Gamba^57^). The activity was measured as the total amount of time spent in motion. We also recorded the number bouts of movements, defined as the number of times an individual was seen moving, a parameter often linked with activity and exploration^25,58^. To assess further the explorative behaviour, the arena was virtually divided with a grid of 69 zones. The exploration was defined as the number of different zones crossed by individuals at least once during the first hour of recording (one hour being enough for the most explorative individuals to cover over 90% of the arena). Finally, the time in inner circle, a behaviour considered as related to both exploration and boldness^20,39^ was measured as the total amount of time during which no part of the individual’s body was touching the arena walls. To avoid any habituation of the testing device that might affect exploration, the second trial of this experiment was conducted 4–5 days after the first one.

### Phototaxis

Single individuals were introduced in 90 mm Petri dishes containing a dark zone (half of the Petri dish being painted in black) and a light zone (artificial light of the maintenance room). After five minutes of habituation in the device, the position of each individual was recorded every 30 seconds for one hour and scored as zero (dark zone) or one (light zone). Summed phototaxis scores for each individual could range from zero (strongly photophobic) to 120 (strongly photophilic). The experiment was conducted twice for each individual, with 24h between the two trials.

### Statistical analyses

None of the seven behavioural parameters (flee duration, flee distance, activity, exploration, number of movements, time in inner circle and phototaxis) met normality and homoscedasticity conditions. We therefore used non-parametric statistics. Spearman’s rank order correlations were calculated between the two tests of each parameter to evaluate the repeatability of the behaviours.

To identify possible associations among the different parameters, defining personality dimensions, we performed an agglomerative cluster analysis, following previous studies on animal personality^24,25,59–61^. After calculating Spearman rank-correlations among the different parameters (average values for the two tests), we computed a matrix of dissimilarity measures (one minus the absolute value of correlation coefficients). We then performed an agglomerative clustering using the “agnes” function of the R-package “cluster”^62^ with Ward’s clustering method^24,25^. Personality dimensions were identified from the inspection of the resulting silhouette plot and dendrogram. Spearman’s correlations were also used to verify the consistency among variables in each personality dimension. Data were analysed using JMP version 10.0.0 software (SAS Institute, Cary, NC, U.S.A.) and R version 3.4.3 software (R Foundation for Statistical Computing).

## Data Availability

The datasets generated during this study are available from the corresponding author on reasonable request.
